# The prognostic impact of lead times in colorectal cancer patients undergoing cytoreductive surgery and HIPEC

**DOI:** 10.1186/s12957-022-02765-1

**Published:** 2022-09-19

**Authors:** Ylva Jansson, Wilhelm Graf, Lana Ghanipour

**Affiliations:** Department of Surgical Sciences, Uppsala University, 751 85 Uppsala, Sweden

**Keywords:** HIPEC, CRS, Peritoneal metastases, Colorectal cancer, Lead times, Survival

## Abstract

**Background:**

National lead time goals have been implemented across Sweden to standardize and improve cancer patient care. However, the prognostic impact of lead times has not yet been studied in patients with colorectal cancer and peritoneal metastases scheduled for cytoreductive surgery and hyperthermic intraperitoneal chemotherapy (CRS + HIPEC).

**Aim:**

To study the correlation between lead times and overall survival and operability.

**Methods:**

One hundred forty-eight patients with peritoneal metastases originating from colorectal cancer and scheduled for CRS + HIPEC from June 2012 to December 2019 were identified using a HIPEC register at Uppsala University Hospital. Data were collected from medical records concerning operability, overall survival, recurrence and time from diagnosis, and decision to operate to the date of surgery. Patients who had neoadjuvant therapy or no malignant cells in the resected specimens were excluded. Statistical calculations were made with the chi-squared test, Cox regression analysis, and log-rank test.

**Results:**

The median age was 66 years (27–82). Ninety-five were women and 53 were men. One hundred six underwent CRS + HIPEC, 13 CRS only, and 29 were inoperable (open-close). No difference in overall survival was seen when comparing patients with lead times ≤ 34 days and ≥ 35 days from the decision to operate at the multidisciplinary conference to the surgery but there was a higher frequency of open-close (*p* = 0.023) in the group with longer lead time. Factors that impacted overall survival were open-close (*p* < 0.001), liver metastases (*p* = 0.003), and peritoneal cancer index score ≥ 20 (*p* < 0.001).

**Conclusion:**

A long lead time from multidisciplinary conference to surgery has no direct impact on overall survival but can result in more cases of inoperability. In a larger cohort, this might translate into decreased survival, and efforts should therefore be made to complete preoperative work up as soon as possible and reduce overall time span. Important factors for survival are related to patient selection and extent of disease.

## Introduction

Median survival for patients with colorectal cancer and peritoneal metastases who undergo cytoreductive surgery and hyperthermic intraperitoneal chemotherapy (CRS + HIPEC) can be prolonged compared to systemic chemotherapy-only treatment [1]. However, this is applicable only to correctly and thoroughly selected patients and some important predictive factors have been identified. The most important are spread of disease to other organs, as well as to the peritoneum, as measured with the Peritoneal Cancer Index (PCI), and the size of residual disease after CRS graded by the completeness of cytoreduction score (CCS) [1–4]. Lead times have been of interest for evaluating and guaranteeing, equal, and best possible care for cancer patients in Sweden. However, there are no studies of the prognostic influence of lead times in patients undergoing CRS + HIPEC for colorectal cancer with peritoneal metastases. In general, the waiting time from multidisciplinary conference (MDC) to surgery varies depending on availability in hospital resources. The objective of this study was to examine whether there is a difference in overall and operability depending on lead times in patients treated with CRS + HIPEC for peritoneal metastases from colorectal cancer.

## Methods

### Patients and data collection

The cohort was identified from a HIPEC register at Uppsala University Hospital. A total of 226 patients with colon or rectal cancer were scheduled for CRS + HIPEC surgery in Uppsala between June 2012 and December 2019. Those with absence of malignant cells in the resected specimens (*n* = 18) and patients who received neoadjuvant chemotherapy (*n* = 60) were excluded, leaving 148 patients in the study.

Clinicopathological data were collected from the patients’ medical records and the HIPEC register regarding date of diagnosis of peritoneal metastases at laparotomy, laparoscopy, biopsy, or verified by radiology; date of decision for surgery at a multidisciplinary conference; date of surgical scheduling at the clinic; age at surgery; gender; preoperative staging with laparoscopy or laparotomy for synchronous or metachronous disease; PCI and CCS; primary tumor histology, peritoneal histology, postoperative morbidity according to Clavien-Dindo classification [5] and mortality.

The following lead times were analyzed: diagnosis of peritoneal metastases to surgery, multidisciplinary conference decision to surgery and scheduling surgery to surgery. Calculations were based on the medians (Table 1), which properly reflect the standard care process for peritoneal metastases at our center using the categories: 0–58 days and > 59 days for diagnosis of peritoneal metastases to surgery, 0–34 days and > 35 days for multidisciplinary conference to surgery, 0–22 days and > 23 days for scheduling surgery to planned CRS-HIPEC.

**Table 1:** Description of lead times (maximum, minimum, median, and mean) in the cohort

Lead times, days to surgery			
Measurement of position	CPM diagnosis-surgery	MDC-surgery	Operation planning-surgery
Median	58.5	34.0	22.0
Mean	78.2	38.6	25.9
Minimum	8.0	3.0	1.0
Maximum	838.0	202.0	96.0

*CPM* colorectal cancer and peritoneal metastases, *MDC* multidisciplinary conference

Information on survival was collected from the Swedish Population Register and all observations were censored as of December 2020.

The primary endpoints were overall survival (OS) measured from date of surgery to date of death from any cause and operability which was defined as ability to achieve radical surgery, thus avoiding an open-close procedure.

This study was approved by Uppsala County’s Ethics Committee (Dnr 2013/203).

### Surgical procedures and HIPEC regimens

CRS was performed according to Sugarbaker’s techniques and principles, where the extent of resection was decided by the distribution of malignancy [6]. PCI score was used to describe the extent of peritoneal metastases [7], and the result of the CRS was documented using CCS [8]. If it was obvious at initial exploration that adequate CRS (CCS 0-1) could not be achieved, only palliative procedures were considered, e.g., stoma, by-pass, or resection in case of actual or imminent bowel obstruction but no CRS or HIPEC was performed—a so-called open and close procedure. This surgical strategy was in large in accordance with general guidelines for CRS and HIPEC treatment [9]_._

HIPEC was performed intraoperatively according to the Coliseum method [10] with intra-abdominal administration of either oxaliplatin (460 mg/m^2^) or irinotecan (460 mg/m^2^) for a duration of 30 min. Alternatively, mitomycin C (30 mg/m^2^) was used for 90 min. The intra-abdominal temperature was 41–43 °C with a continuous flow of 1–2 L/min. When oxaliplatin or irinotecan was used, a single bolus of IV 5-FU, 400 mg/m^2^, was given intraoperatively and folinic acid, 60 mg/m^2^, 30 min before the administration of HIPEC. Thirteen patients were treated with CRS without HIPEC mainly because of intraoperative difficulties (severe adhesions, complicated reconstructions, etc.). HIPEC was performed only if adequate CRS could be obtained. A total of 16 patients underwent concomitant resection of hepatic metastases.

### Histopathological variables

Proto oncogene mutation status (KRAS and BRAF) was assessed by pyrosequencing and was performed selectively based on clinical indications [11]. All tumors showing any amount of signet ring cells were counted as “presence of signet ring cells” [12].

### Statistical analysis

Differences in proportions between groups were evaluated using the chi-squared test. Hazard ratio, with a 95% confidence interval (CI), were calculated with Cox regression analysis in univariate and multivariate models. Survival was analyzed with the Kaplan-Meier method, and the log-rank test was used as test of significance. Statistical significance was set at *p* < 0.05. For statistical analyses, SPSS statistics version 27 (SPSS Inc. Chicago, IL. USA) was used.

## Results

### Patient characteristics

Of the 148 included patients, 95 were women and 53 were men. The median age was 66 years [27–82]. One hundred and six underwent CRS + HIPEC, 13 CRS only, while 29 were open-close (Table 2). The most common primary tumour site was the right colon. One patient presented with synchronous tumors in the rectum and right colon. Adjuvant systemic chemotherapy after CRS + HIPEC was given postoperatively to 58 (39%) of the patients (Table 2). The decision to recommend adjuvant systemic chemotherapy was mainly based on features of the histopathology of the specimen.

**Table 2:** Clinicopathological factors in patients with peritoneal metastases from colorectal cancer

Factor	***n*** = 148	%
**Age (median 66 years)**		
< 66 years	73	49.3
≥ 66 years	75	50.7
**Gender**		
Women	95	64.2
Men	53	35.8
**Timing of CPM**		
Synchronous CPM	73	49.3
Metachronous CPM	75	50.7
**Location of primary tumour**		
Rectum	15	10.1
Left colon	52	35.1
Right colon	80	54.1
Data missing	1	0.7
**Preoperative laparotomy or laparoscopy**		
Yes	53	35.8
No	95	64.2
**Reason for prolonged time to decision for surgery**		
Tumor investigation	142	95.9
Medical investigation of patient factors	1	0.7
Other	5	3.4
**Type of surgery**		
CRS + HIPEC	106	71.6
CRS	13	8.8
Open-close	29	19.6
**HIPEC regime**		
Oxaliplatin	89	59.5
Irinotecan	14	9.5
Mitomycin C	3	2
**PCI score**		
PCI ≤ 20	102	68.9
PCI ≥ 21	40	27
Data missing	6	4.1
**Cytoreduction score**		
CC0	106	71.6
CC1	11	7.4
CC2	1	0.7
CC3	29	19.6
Data missing	1	0.7
**Clavien-Dindo grade**		
Grade I–II	109	73.6
Grade III–IV	37	25
Dead	2	1.4
**Liver metastases**		
Yes	16	10.8
No	132	89.2
**Signet ring cells**		
Yes	28	18.9
No	118	79.7
Data missing	2	1.4
**BRAF mutation**		
Yes	10	6.8
No	62	41.9
ND	76	51.4
**KRAS mutation**		
Yes	35	23.6
No	38	25.7
ND	75	50.7
**Adjuvant treatment**		
Yes	58	39.2
No	87	58.8
Data missing	3	2

*CPM* colorectal cancer and peritoneal metastases, *ND* not done

A major cause of prolonged time to surgery was preoperative tumour-related investigations (Table 2).

Mean time to operation measured from the date of peritoneal metastases to diagnosis was 58.5 days, from multidisciplinary conference 34 days, and from scheduling surgery 22 days (Table 1).

Neither age, gender, liver metastases, synchronous or metachronous metastasis, PCI ≥ 20, TN-stage, primary tumour location, mucinous tumors, grade of differentiation, whether adjuvant treatment had been given, nor the presence of KRAS or BRAF mutations were significantly associated with lead time from diagnosis of peritoneal metastases to surgery. There were no correlations between longer lead times from scheduling the operation to the surgery with any of the factors studied either.

There was a significant correlation between lead times from multidisciplinary conference to surgery and the presence of signet ring cells (*p* = 0.041) as well as open-close procedures (*p* = 0.023) (Table 3). Age, gender, synchronous or metachronous metastases, preoperative laparotomy or laparoscopy, PCI ≥ 20, TN-stage, primary tumour location, mucinous tumors, grade of differentiation, and the presence of KRAS or BRAF mutations were also analyzed but none was significantly associated with lead time.

**Table 3:** Variables with significant correlation with lead times from multidisciplinary conference to surgery when divided into two groups (≤ 34 days and ≥ 35 days)

Factor	≤ 34 days		≥ 35 days		
	***n*** = 79	%	***n*** = 69	%	***p-*** value
Open-close					
Yes	10	12.7	19	27.5	0.023
No	69	87.3	50	72.5	
Signet ring cells					
Yes	20	25.3	8	11.6	0.041
No	59	74.7	59	85.5	
Missing data	0	0	2	2.9	
Liver metastases					
Yes	5	6.3	11	15.9	0.060
No	74	93.7	58	84.1	

### Survival analysis

**Fig. 1:**
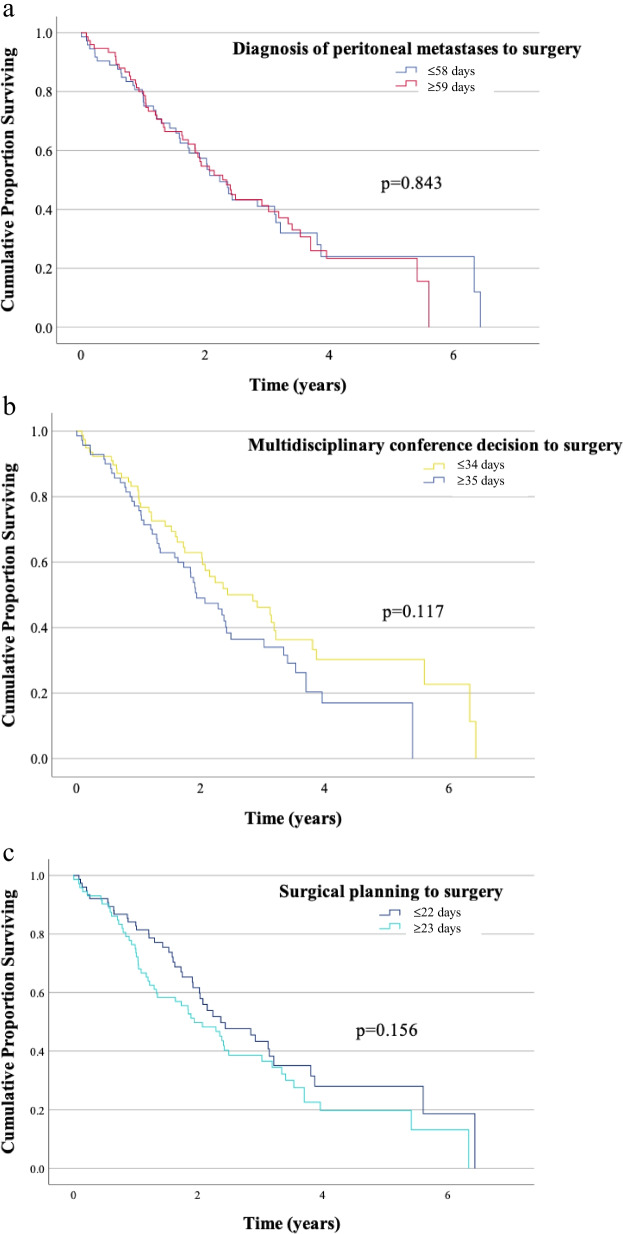
Kaplan-Meier curve illustrating overall survival related to lead time from date of diagnosis of peritoneal metastases to surgery (**a**), date of multidisciplinary conference decision to surgery (**b**) and date of scheduling surgery (**c**)

No significant difference in overall survival was seen when analyzing lead times in univariate- and multivariate cox regression analyses (Table 4) and when compared with the Kaplan-Meier method (Fig. 1a–c).

**Table 4:** Univariate and multivariate Cox proportional analysis of factors affecting overall survival in patients with peritoneal metastases from colorectal cancer

Factor	Univariate		Multivariate	
	HR (95% CI)	***p*** value	HR (95% CI)	***p-*** value
Liver metastases				
No	1 (ref)		1 (ref)	
Yes	1.88 (1.04**–**3.39)	0.037	2.52 (1.36**–**4.65)	0.003
PCI-score				
≤ 20	1 (ref)		1 (ref)	
≥ 20	3.94 (2.53**–**6.14)	< 0.001	2.61 (1.53**–**4.44)	< 0.001
Signet ring cells				
No	1 (ref)		1 (ref)	
Yes	2.16 (1.33**–**3.50)	0.002	0.67 (0.40**–**1.14)	0.140
Open-close				
No	1 (ref)		1 (ref)	
Yes	5.99 (3.67**–**9.73)	< 0.001	4.16 (2.30**–**7.50)	< 0.001
MDC decision to surgery				
≤ 34 days	1 (ref)			
≥ 35 days	1.34 (0.89**–**2.02)	0.163		
CPM diagnosis to surgery				
≤ 58 days	1 (ref)			
≥ 59 days	1.04 (0.69**–**1.57)	0.843		
Time period				
June 2012–December 2016	1 (ref)			
January 2017–December 2019	0.79 (0.52**–**1.21)	0.285		

*MDC m*ultidisciplinary conference, *CPM* colorectal cancer and peritoneal metastases

Univariate Cox regression analysis showed that there was a correlation between the increased risk of dying and liver metastases, PCI ≥ 20, presence of signet ring cells, and open-close (Table 4). Multivariate analysis showed a correlation between open-close (*p* < 0.001), PCI ≥ 20 (*p* < 0.001) and liver metastases (*p* = 0.003) and short overall survival (Table 4). To detect changes over time and the impact of early or late treatment, two separate time periods were analyzed, which showed no effect on overall survival (Table 4).

### Postoperative morbidity and mortality

Clavien-Dindo Grade III-IV was registered in 37 (25%) of the patients, of whom 12 were re-operated due to anastomotic leakage (*n* = 3), bleeding (*n* = 3), bowel perforation (*n* = 3), bladder perforation (*n* = 1), and small intestine ischemia (*n* = 1). There were two deaths within 30 days.

## Discussion

No significant difference in overall survival depending on lead times was shown. On the other hand, there was a relationship with higher frequency of open-close and a time ≥ 35 days from MDC decision to surgery (*p* = 0.023). This is new information within this scientific field. At the same time, three factors correlated with a higher risk of death. These were open-close, PCI ≥20 and liver metastases. Liver metastases tended to be more frequent in the group with longer times from MDC to surgery, whereas PCI showed no significant correlation with longer lead times. The most common reason for postponing surgery was a tumor-related investigation such as PET, MRI scans, or biopsies. Hence, more complex tumors could be overrepresented in the group with longer lead times.

No previous studies have investigated the impact of lead times for patients with peritoneal metastases from colorectal cancer undergoing CRS + HIPEC. These patients have a relatively short median survival time both after treatment with systemic chemotherapy (approximately 16 months) as well as after CRS + HIPEC (42 months) [1]. Furthermore, no other factors except open-close and presence of signet ring cells had a significant correlation with longer lead times.

Nonetheless, it is not possible to rule out an uneven distribution of unknown confounders in our study, which might have influenced the results. There are data suggesting an association with worse short- and long-time survival for all lead times longer than 6 weeks [13]. Other findings suggest that, on a population level, there is no relationship between time to surgery and survival, but that there is when including factors such as age, comorbidities, and stage of cancer [14–16]. One study shows that both extremes (very short and long lead times) are associated with shorter survival. However, short waiting times are believed to be associated with more emergency presentations and severe disease. Comorbidities were not entirely investigated either [17].

Postoperative complications were not more common in the groups with longer lead times. It can be noted that the severity and prevalence of postoperative complications in our cohort were similar, or even lower, compared with previous reports [18]. This is interpreted as an indication that patients should be thoroughly investigated before having CRS + HIPEC since there is high risk for complications related to the surgery.

The fact that the open-close procedure was correlated with an increased risk of death is noteworthy, as there was also a correlation with a longer time from the multidisciplinary conference decision to surgery, but at the same time no difference in survival for the same group. One possible reason could be that the efficacy of systemic treatment has greatly improved in recent years and is therefore now a good alternative for prolonging life in inoperable patients [19]. Another alternative is that the population in our study was too small to find a correlation with survival. However, the fact that there was a difference in the frequency of open-close procedures might be an indication that there is an actual difference, albeit not visible in this study.

Several studies have investigated exactly what it is that affects survival and to what extent. In our study, three different factors, all of which have been noted previously as affecting survival, were significant in multivariate analysis: liver metastases [12], open-close, and PCI ≥ 20 [2, 12]. Other factors were also shown to be important for prognosis. One example is CCS which was not included in the multivariate analysis since this variable is strongly correlated to open-close [2, 12].

The inexact counting of signet ring cells made it impossible to draw any firm conclusions about signet ring cell adenocarcinoma. At the same time, there is evidence that even a smaller component of signet ring cells is correlated with an increased risk of developing peritoneal metastases in general [20] and shorter survival after CRS + HIPEC [21]. It is possible that tumours with very few signet ring cells were included in our study and that they are less aggressive, but there was no information in our registers about the exact proportion of signet ring cells.

Patients who were not considered representative were excluded. Neoadjuvant treatment prolongs lead times by necessity, but it is not the standard strategy in Uppsala or elsewhere in Sweden [22]. Moreover, patients who had no malignant cells in the resected peritoneum cannot be considered to have peritoneal metastases and are therefore not relevant to the study. The other factors examined were chosen for analysis if they had been identified as relevant to the prognosis and were present in the medical records.

Several aspects were taken into consideration when dividing the cohort into different groups to investigate lead times. The goal was to create groups of the same size, based on medians. At the same time, the medians reflected national goals and realistic clinical expectations about acceptable delays to definite treatment.

It is the Swedish tradition to strengthen the trustworthiness of any medical methodology by thorough mapping and data collection of patients in various registers. Our study was able to take advantage of this. The risk for unknown confounders is also low in our study since all patients underwent surgery in Uppsala. Subsequently, differences in surgical methods, as well as in pre- and postoperative care, should be minimal due to local standardization.

Weaknesses in our study include limitations in information registered in the medical records. This was primarily seen as missing data for some patient and tumor-related variables.

Furthermore, one could question whether the quantitative approach of this study lacks nuance as it does not take into consideration the patients’ own experiences of waiting, or how this in turn affects mental health. Future qualitative studies would be warranted in this respect.

## Conclusions

There was no definite correlation between longer lead times and survival. However, longer lead times increased the risk for open and close surgery. With a larger patient cohort, this might translate into decreased survival. Thorough patient selection for CRS + HIPEC is of the greatest importance since morbidity is high with this procedure. We conclude that, today, there is not enough evidence that the time to surgery needs to be shortened at the cost of a deficient preoperative work up. A certain amount of time is needed to investigate the patient, since advanced tumor stage require more extensive investigations, and therefore constitute the most common reason to prolonged lead times.

Further studies would be justified in the future, to obtain more reliable results, including whether results are applicable to other HIPEC centra, as well as the population at large, both nationally and globally. The same applies to mapping those additional prognostic factors that can further improve the selection of patients for surgery and oncological treatment.

## Data Availability

The data underlying the results presented in this study contain potentially sensitive and identifying participant information and cannot be shared publicly due to GDPR. However, the raw data are available upon reasonable request directed to the corresponding author.
